# Evolutionary patterns of proteinase activity in attine ant fungus gardens

**DOI:** 10.1186/1471-2180-11-15

**Published:** 2011-01-19

**Authors:** Tatyana A Semenova, David P Hughes, Jacobus J Boomsma, Morten Schiøtt

**Affiliations:** 1 Centre for Social Evolution, Department of Biology, University of Copenhagen, Universitetsparken 15, DK-2100 Copenhagen, Denmark; 2 Department of Ecology and Agriculture, University of Copenhagen, Thorvaldsensvej 40, DK-1871 Frederiksberg C, Denmark; 3 A.N. Belozersky Institute of Physico-Chemical Biology, Moscow State University, Leninskie Gory 1, Moscow 119992, Russia; 4 Current address: Museum of Comparative Zoology, Harvard University, 26 Oxford Street, Cambridge MA 02138, USA

## Abstract

**Background:**

Attine ants live in symbiosis with a basidiomycetous fungus that they rear on a substrate of plant material. This indirect herbivory implies that the symbiosis is likely to be nitrogen deprived, so that specific mechanisms may have evolved to enhance protein availability. We therefore hypothesized that fungal proteinase activity may have been under selection for efficiency and that different classes of proteinases might be involved.

**Results:**

We determined proteinase activity profiles across a wide pH range for fungus gardens of 14 Panamanian species of fungus-growing ants, representing eight genera. We mapped these activity profiles on an independently obtained molecular phylogeny of the symbionts and show that total proteinase activity in lower attine symbionts peaks at ca. pH 6. The higher attine symbionts that have no known free-living relatives had much higher proteinase activities than the lower attine symbionts. Their total *in vitro *proteinase activity peaked at pH values around 5, which is close to the pH that the ants maintain in their fungus gardens, suggesting that the pH optimum of fungal proteinases may have changed after the irreversible domestication of evolutionary more derived fungal symbionts. This notion is also supported by buffering capacities of fungus gardens at pH 5.2 being remarkably high, and suggests that the fungal symbiont actively helps to maintain garden acidity at this specific level. Metalloproteinases dominated the activity profiles of lower attine gardens and may thus represent the ancestral type of proteinase production, whereas serine proteinase activity dominated the activity profiles of the higher attine gardens reared by *Trachymyrmex *and *Sericomyrmex*, suggesting that there may be trade-offs in the production of these enzyme classes. Remarkably, the single symbiont that is shared by species of the crown group of *Atta *and *Acromyrmex *leaf-cutting ants mostly showed metalloproteinase activity, suggesting that recurrent changes in enzyme production may have occurred throughout the domestication history of fungus-garden symbionts.

**Conclusions:**

Proteinase pH optima and buffering capacities of fungal symbionts appear to have evolved remarkable adaptations to living in obligate symbiosis with farming ants. Although the functional roles of serine and metalloproteinases in fungus gardens are unknown, the differential production of these classes of proteolytic enzymes suggest that substrate specificity may be important and that trade-offs may prevent the simultaneous upregulation of both classes of enzymes.

## Background

Organisms that engage in an obligate mutualistic lifestyle often experience a drastic change in environmental conditions. Well known examples are symbiotic bacteria in the rumen of ungulates and the mitochondria in eukaryotic cells, which function under quite different growth conditions than free-living bacteria, and have genomes that became modified or reduced in response to these specialized dependent life styles [1,2]. However, the expression of derived symbiotic traits is difficult to study in endosymbiotic bacteria, because they can normally not be grown on artificial media [3] or otherwise be studied separately from the host. This is easier in obligate ectosymbioses where hosts and symbionts can often survive and function without their partner-mutualist for at least a short period, and where relatively pure samples of symbiont biomass can often be obtained and analyzed.

Attine ants live in obligate mutualistic association with specific fungi that they rear for food in underground gardens. The cultivated fungi mostly belong to the tribe *Leucocoprini *(Basidiomycotina: Agaricales: Agaricaceae) [4,5] which primarily consists of free-living saprotrophic genera that grow in the lower litter layer of forest floors, usually characterized by high pH levels [6] The ants supply their mutualistic fungi with substrate and protect their gardens from infections [5]. One of the defense mechanisms to control diseases is the secretion of the ant's metapleural glands [7-10], which generates acidic conditions in fungus gardens, discouraging microbial growth relative to the surrounding soil with higher pH. Acetic acid is being produced in the fungus gardens, but this has to be tightly regulated as it has the potential to inflict more harm to the symbiont than to alien fungi [10]. The domestication of fungal symbionts by the ants may therefore have required specific adaptations for growing under lower but more controlled pH conditions, both in terms of pH buffering and with regard to the activity optima of the extracellular enzymes that are produced to degrade the substrate that the ants collect.

Nitrogen is a limiting factor for growth and maintenance in many organisms, particularly those living on a herbivorous diet as the attine ants indirectly do. Recent findings show that leaf-cutting ants partly overcome nitrogen limitation by living in association with N_2_-fixing bacteria that may supply as much as 50% of a colony's nitrogen requirements [11]. Such bacterial nitrogen will be incorporated into proteins, so that the fungal symbionts of the ants must secrete proteinases to digest these into amino acids that can be assimilated. The fungal symbiont is also likely to compete for nitrogen with other, non mutualistic microorganisms living in the fungus garden [12,9,13], imposing further selection for effective protein degradation by the fungal symbiont. Finally, proteolytic enzymes are known to be strongly pH dependent, so in order to have effective protein degradation the pH optimum of the proteolytic enzymes should ideally match the pH of the fungus garden.

Several studies have been devoted to the role of pH in controlling *in vitro *proteolytic enzyme secretion in fungi [14], but to our knowledge *in vivo *studies of pH-dependent proteolytic enzyme activities in fungi have not been done. The objective of our present study was thus threefold: 1. To use the unique growth form of ant fungus gardens to determine the feasibility of pH buffering studies in fungi, 2. To determine the pH activity optima of different classes of extracellular proteinases across a series of genera and species of fungus-growing ants, and 3. To map the observed differences on an independently obtained phylogenetic tree of the fungal symbionts to obtain insight in the evolutionary pathways that may have generated differences in pH-dependent activities of proteinases.

## Results

### The pH conditions of fungus gardens and their buffering capacity

All 29 attine ant colonies used in this study (see Table 1 for details) displayed the same pH (5.2 ± 0.1) for 1:1 water extracts taken from the middle layer of the fungus gardens. Adding acid/alkaline solutions to the fungus garden extracts did not noticeable change the color of pH paper compared to controls (data not shown) indicating that all tested fungus gardens exhibited approximately the same buffering strength.

**Table 1:** Total and class-specific relative proteolytic activity and its pH optimum range measured in fungus gardens.

Ant species	Colony number	Sample number	Total activity	pH optimum	Metallo-proteinase activity	pH optimum	Serine proteinase activity	pH optimum	Aspartic proteinase activity	Cysteine proteinase activity
*Apterostigma collare*	Apcol1	-	630.0 ± 18.3		593.0 ± 13.3		1.7 ± 0.5		16.0 ± 1.0	0.8 ± 0.5

*Myrmicocrypta ednaella*	Myred1	1	168.6 ± 9.5	6.2 ± 0.11	151.5 ± 6.4	6.0 ± 0.04	9.4 ± 1.0	7.0 ± 0.012	--	9.3 ± 1.0
	Myred2	2	165.2 ± 9.2		104.0 ± 5.0		50.1 ± 6.3		--	--

*Mycocepurus smithii*	Mycsmi9	3	114.0 ± 9.0	6.0 ± 0.11	101.6 ± 4.8	6.0 ± 0.1	5.3 ± 1.0		4.1 ± 1.0	3.6 ± 1.0
	Mycsmi15	4	136.6 ± 9.6		124.5 ± 8.7		6.7 ± 1.0		--	--
	Mycsmi32	5	153.0 ± 10.7		148.7 ± 8.5		2.8 ± 1.0		1.3 ± 1.0	--

*Cyphomyrmex costatus*	Cycos6	6	65.2 ± 8.2		54.8 ± 5.0		5.9 ± 2.0		1.6 ± 1.0	1.6 ± 0.8
	Cycos9	7	61.3 ± 5.0	6.0 ± 0.11	47.4 ± 4.5	6.0 ± 0.08	3.3 ± 1.0		3.1 ± 1.0	3.7 ± 1.0
	Cycos16	8	112.5 ± 9.0		90.8 ± 4.3		19.0 ± 3.2		2.8 ± 1.0	--

*Cyphomyrmex longiscapus*	Cylon12	9	131.5 ± 8.7	6.0 ± 0.09	106.9 ± 7.5	6.0 ± 0.1	18.9 ± 2.0		3.2 ± 1.0	3.2 ± 1.1
	Cylon5	10	140.6 ± 9.8		131.0 ± 5.2		6.4 ± 2.0		3.7 ± 1.0	--
	Cylon24	11	146.5 ± 9.0		132.5 ± 9.0		6.6 ± 2.4		5.2 ± 1.4	--

*Sericomyrmex amabilis*	Serama8	12	210.0 ± 8.9	5.2 ± 0.015	48.1 ± 4.4	5.0 ± 0.1	108.1 ± 5.6	7.0 ± 0.075	30.0 ± 10.2	29.0 ± 6.4
	Serama7	13	194.1 ± 12.4		22.3 ± 3.5		130.5 ± 6.3		30 ± 8.8	26 ± 7.2
	Serama12	14	308.1 ± 9.0		42.5 ± 4.2		227.1 ± 9.9		21.1 ± 7.4	23.4 ± 5.2

*Trachymyrmex cornetzi*	Trcor1	15	310.3 ± 10.3		262.9 ± 9.1		49.4 ± 4.0		--	3.2 ± 1.0
	Trcor3	16	333.4 ± 9.5		211.5 ± 7.4		46.1 ± 4.2		--	78.0 ± 5.5
	Trcor4	17	257.4 ± 9.2	5.7 ± 0.07	92.4 ± 7.2	6.05 ± 0.1	138.4 ± 8.3	5.7 ± 0,1 7.5 ± 0.05	5.0 ± 1.3	22.1 ± 4.6
	Trcor10	18	155.0 ± 9.6	5.7 ± 0.07	131.9 ± 7.12	5.7 ± 0.09	7.7 ± 1.0		7.14 ± 2.1	7.15 ± 1.1

*Trachymyrmex sp. 3*	Trsp3-3	19	201 ± 9.1	5.2 ± 0.11	35.0 ± 9.8	5.7 ± 0.09	153.1 ± 10.42	7.5 ± 0.09 5.2 ± 0.09	7.0 ± 1.5	8.4 ± 2.2
	Trsp3-6	20	249.7 ± 9.4		33.5 ± 7.4		199.2 ± 9.0		--	20.0 ± 7.8

*Trachymyrmex zeteki*	Trzet2	21	340.1 ± 11.0		67.4 ± 5.0		215.5 ± 7.5		--	55.7 ± 8.8
	Trzet3	22	342.3 ± 9.5	5.2 ± 0.1	28.4 ± 7.0	5.2 ± 0.09	317.0 ± 7.1	5.35 ± 0.08	--	--
	Trzet6	23	340.1 ± 8.9		70.6 ± 6.0		261.5 ± 9.0		1.39 ± 1.5	6.5 ± 1.3

*Acromyrmex echinator*	Acech322	24	323.3 ± 10.0	5.4 ± 0.11	227.5 ± 10.6	5.2 ± 0.09	66.5 ± 6.4	7.5 ± 0.06	18.5 ± 6.3	--

*Acromyrmex octospinosus*	Acoct1	25	454.2 ± 15.2		322.1 ± 12.5		64.2 ± 5.5		--	56.2 ± 6.0

*Atta colombica*	Atcol1	26	332.1 ± 14.8		227.5 ± 10.5		66.5 ± 6.02		18.5 ± 4.6	--

*Atta sexdens*	Atsex1	27	390.0 ± 13.5		300.6 ± 11.6		35.7 ± 9.0		18.4 ± 6.3	40.1 ± 5.4

*Atta cephalotes phalotes*	Atcep1	28	300.1 ± 14.7		193.1 ± 13.06		30.1 ± 6.41		35.5 ± 4.9	50.1 ± 6.6

One unit of relative proteolytic activity (U) corresponds to 1*10^(-3) ^difference between treatment and control absorbance (A_440_, at t°C 26°C, 1 hour). The mean activity values are presented as U*10^3 ^± SE.

Approximate buffering capacities for attine symbionts and non-symbiotic basidiomycetous fungi (Table 2) showed that fungus garden symbionts and plated *L. gongylophorus *(ca 6 - 22 mekv/L) tended to have higher buffering capacities than free-living fungi (ca 2 - 10 mekv/L) (t_23 _= -8.6, p < 0.001). The buffering capacity of plated fungi did not depend on the composition of the cultivation media. The relationship between the pH and the amount of added base was approximately linear (R^2 ^not less than 0.976, data not shown) suggesting that all measurements were performed in the pH zone close to the buffer point of the tested solutions where they exhibit their maximal buffering capacity [15].

**Table 2:** Buffering capacity (means ± SE in mekv/L) for free living fungi and fungus garden symbionts of attine ants.

Fungal species (family)	Buffering capacity, mekv/L	Sample size
*Free-living fungi, plated*		
*Agaricus bisporus *(Agaricaceae)	9.6 ± 1.08 (strain 1)	5
	7.3 ± 0.92 (strain 2)	5
*Pleurotus ostreatus *(Pleurotaceae)	4.95 ± 0.7	5
*Pleurotus pulmonarius *(Pleurotaceae)	3.1 ± 0.12	5
*Lentinula edodes *(Marasmiaceae)	2.01 ± 0.1	5

*Fungus garden symbiont, plated*		
*Leucocoprinus gongylophorus *(Agaricaceae)	16.2 ± 2.01	3

*Fungus garden symbiont*, *colony*		
*Apterostigma collare*, (Apcol1)	not measured*	
*Myrmicocrypta ednaella*, (Myred2)	21.92	3
*Mycocepurus smithii*, (Mycsmi32)	21.89	3
*Trachymyrmex cornetzi*, (Trcor1)	20.55	3
*Sericomyrmex amabilis*, (Serama7)	16.74	3
*Sericomyrmex amabilis*, (Serama12)	5.80**	3
*Acromyrmex echinator*, (Acech322)	17.93 ± 1.54	3
*Acromyrmex octospinosus*, (Acoct1)	16.80	3
*Atta colombica*, (Atcol1)	17.64	3
*Atta cephalotes*, (Atcep1)	22.20	3

* Buffering was observed on pH test papers only, but was comparable to the other fungal garden symbionts.

** This colony of *Sericomyrmex amabilis *(Serama12) had an unusually solid and humid garden structure compared to all other fungus gardens examined.

### Differential production of proteinase classes across fungus gardens

All tested colonies displayed significant proteinase activity (Table 1). The mean total activity values ± SE were 127 ± 11, 270 ± 19 and 360 ± 28 U·10^3 ^(± SE) for lower attine, higher attine and leaf-cutting ant gardens, respectively, which implies that total proteinase activity increases with the degree of evolutionary "advancement" of the symbiosis. However, the garden of *Apterostigma collare *was an exception to this rule, expressing relatively high total proteinase activity compared to the other lower attine ants. This is remarkable as these ants rear a phylogenetically distant fungus, belonging to the family *Pterulaceae*, while all other attines cultivate fungi belonging to the *Leucocoprini *tribe of the family *Agaricaceae *[4,5].

Inhibition analyses revealed that proteinases belonging to all four catalytic classes could be detected in the fungus gardens (Table 1), but the activity of aspartic and cysteine proteinases was very low compared to the activity of serine- and metalloproteinases. This result was not unexpected as cysteine and aspartic proteinases are rarely produced by fungi [16,17]. The serine proteinases belonged to the subtilase-like superfamily as they were inhibited by PMSF, but not by TLCK and TPCK [18], and they displayed activity towards the chromogenic substrates Glp-AAL-pNa and Suc-AAPF-pNa, but not to N-benzoyl-Arg-pNa [19]. The metalloproteinases could not be further identified.

**Figure 1:**
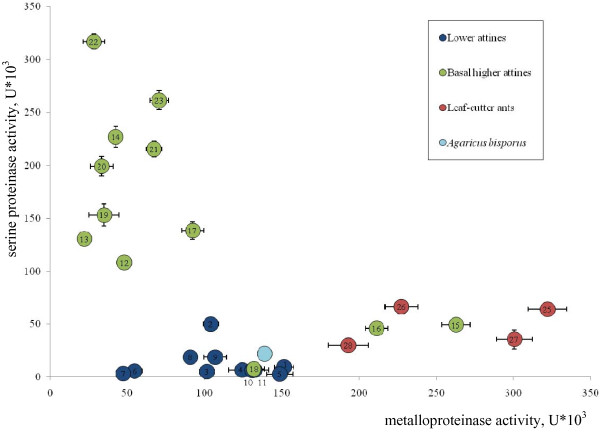
**Fungal proteolytic activity (see Table 1) partitioned between serine- and metalloproteinases. **Lower attine, basal higher attine and leaf-cutting ant activities are plotted in blue, green and red, respectively.

The activities of the two dominant classes of proteinases varied considerably between fungus gardens (Figure 1), with lower attine symbionts having low activities for both classes and the basal higher attine- and leaf-cutting symbionts specializing in producing mostly only one class. Leaf-cutting ant gardens were characterized by high activity of metalloproteinases, similar (at least in relative activity) to the lower attine gardens, whereas the gardens of basal higher attine ants, with one exception, primarily showed serine proteinase activity (Figure 1).

### Mapping proteolytic activity profiles on the phylogenetic tree of the fungal symbionts

**Figure 2:**
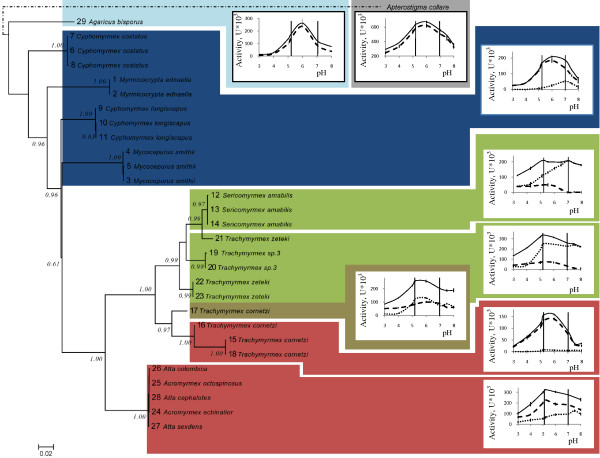
**pH-dependent proteolytic enzyme activity profiles mapped on the fungal symbiont phylogeny. **The pH optima curves concern total proteinase activity (solid lines) and metallo- and serine proteinase activity separately (dashed and dotted lines, respectively). Vertical lines on the graphs represent the respective pH conditions of fungus gardens (5.2) and the typical pH optimum for alkaline proteinases (7.0). The profiles of lower attines plus higher attines with mainly serine proteinase activity and higher attine and leaf-cutting ants with mainly metalloproteinase activity are outlined with blue, green and red backgrounds, respectively, to match color-coding in Figure 1. The single *Trachymyrmex cornetzi *garden with an intermediate proteinase profile is plotted against a brown background and the single *Apterostigma collare *colony rearing a pterulaceous fungal symbiont against a grey background. The numbering of fungus gardens corresponds to the numbers used in the Table 1. The *Myrmicocrypta ednaella *(Myred1) profile is representative for all lower attine gardens. The *Sericomyrmex amabilis *(Serama1) garden was chosen as illustrative example because it showed a relatively high metalloproteinase activity, so that a clearer pH profile could be obtained. For *Trachymyrmex *species with predominantly serine proteinase activity we plotted the average profile for *Trachymyrmex sp3 *(Trsp3-3) and *Trachymyrmex *cf. *zeteki *(Trzet3), which were very similar. As representatives of the basal higher attine and leaf-cutting ant symbionts with predominantly metalloproteinase activity we plotted gardens of colonies Trcor10 and Acech322 as gardens of other colonies with this symbiont displayed very similar profiles. The phylogenetic tree is based on the LSU rRNA and Elongation Factor 1-alpha genes, except for samples 20 and 23 for which only the LSU gene could be sequenced. Only aLRT (approximate likelihood ratio test) support values > 0.5 are given.

Mapping the pH optima curves of proteinase activity on the phylogenetic tree of the fungal symbionts (Figure 2) showed distinct correlations between symbiont clades and the classes of proteinases that were primarily active. High serine proteinase activity was typical for the symbionts of *Sericomyrmex amabilis*, *Trachymyrmex sp3*, and *T. *cf. *zeteki*, which formed a monophyletic group. In contrast, the symbionts of *T. cornetzi *had a proteinase profile resembling that of the *Acromyrmex *and *Atta *leaf-cutting ants, and formed a sister group to the remaining *Trachymyrmex *and *Sericomyrmex *symbionts. The only exception to this pattern was one of the four symbionts of *T. cornetzi *(Trcor4), which had an intermediate proteinase profile with almost equal serine- and metalloproteinase activity, and which formed the most basal branch of the *T. cornetzi *clade of symbionts (number 17, Figure 2).

**Figure 3:**
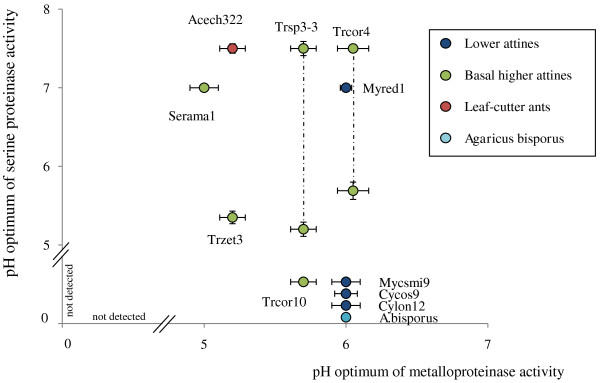
**The class-specific pH optima for serine (vertical axis) and metalloproteinases (horizontal axis) for the fungus gardens in Table 1 for which both pH optima could be measured from the same samples. **The vertical axis is interrupted to allow the pH-optimum to be plotted for metalloproteinase activity in gardens where serine proteinase activity could not be measured. The overall pattern indicates that pH optima for metalloproteinases are always between ca. 5 and 6, whereas serine proteinase pH optima tend to fall between 7 and 8. All values are means ± SEMs. Dotted lines connect observations for the same species. See Table 1 for details.

The pH optimum of total proteinase activity in the gardens reared by lower attines averaged 6.0 ± 0.11, while the peak of proteinase activity in basal higher attines and leaf-cutting ants colonies was closer to the pH levels (ca. 5) in the fungus gardens (Figure 3; Table 1) (t_36 _= 9.3, p < 0.001), as one would expect when the higher attine fungus gardens would have become adapted to growing under more acidic conditions. However, in three of the four clades of higher attine and leaf-cutting symbionts, the total proteinases activity profiles between pH 5 and 7 were remarkably flat, as serine proteinases became increasingly active at higher pHs (Figures 2 and 3). For example, the serine proteinases in *Sericomyrmex amabilis *colonies were most active at pH conditions of 7.0 ± 0.05, similar to lower attine gardens (7.0 ± 0.09) where serine proteinase activity is rarely seen, whereas an additional peak of serine proteinase activity at pH 5.2 ± 0.11 (different from the 7.0 mean above: t_6 _= 17.0, p < 0.001) was observed for the symbionts of *Trachymyrmex sp3 *and *T*. cf. *zeteki*, but not for *Sericomyrmex *symbionts (Figures 2 and 3 and Table 1). Similar patterns were observed for metalloproteinases. The relatively low amounts produced in the symbionts of lower attine ants were most active under slightly acidic pH conditions (6.0 ± 0.11) and shifts towards more acidic optima were detected for the symbionts of *Trachymyrmex cornetzi *(5.6 ± 0.09) (t_4 _= 3.45, p = 0.026) and the leaf-cutting ants mutualists (5.2 ± 0.08) (t_6 _= 10.0, p < 0.001) (Figures 2 and 3 and Table 1).

## Discussion

### Fungus-garden pH buffering capacity is remarkably high

Acidification of the environment increases the difference between the extracellular surroundings and the intracellular milieu, so that compensating metabolic activities are required to maintain the pH within cells at the approximately neutral values that normally optimize growth and survival of fungal hyphae [20]. *In vitro*, these metabolic activities include the synthesis of pH regulating compounds and the modification of excreted compounds so they can function under acidic conditions [21,14,23]. This is particularly important for the extracellular proteolytic enzymes secreted by the fungal symbiont of the leaf-cutting ants, because these enzymes secure the decomposition of proteins that ultimately supply nitrogen to the ant colony [24,25].

Fungi are known to modify the environmental pH *in vitro *[14] and to regulate pH *in vivo *by secreting weak organic acids [23] with buffering properties [26,27]. However, fungi normally avoid natural habitats with unsuitable pH [6], possibly because of the metabolic costs of this type of adjustments in competition with more specifically pH-adapted microorganisms. This may explain why there are only few documented examples of active pH adjustment by organic acid production in free-living fungi [21,23] and to our knowledge no active pH regulation by alkaline production has ever been observed in fungi. This implies that the pH-buffering characteristics of attine fungus gardens are relatively unique. Although the chemistry of the garden buffering mechanism is unknown, its value of ca. 20 mekv/L is comparable to the pH buffering capacity of human blood (37 mekv/L; [28]) and much higher than any value observed outside metazoan bodies - cf. ocean water with 2.4 mekv/L [29] or soil with 2.2 mekv/L [30].

Although the production and secretion of buffering agents may impose significant metabolic costs, this may be sustainable because domestication implies that the ants provision the fungus with ad libitum resources. The benefits of buffering at a constant pH of ca. 5.2 might then be that this value represents a compromise between enhancing efficiency of degradation enzymes and discouraging the growth of parasitic microorganisms that infect fungus gardens [10,31]. If such dynamic equilibrium would exist, it might imply that acidification by the ants and/or the symbiont can be maintained continuously because pH-buffering ensures the necessary stability required for vital fungus garden functions. It seems unlikely that fungal buffering compounds are primarily targeted towards neutralizing the antimicrobial metapleural gland secretions of pH 2.5 [9,10], as a recent study has shown that the ants apply these secretions in very small portions and with great care [32]. The main cause of fungus gardens acidification thus remains unknown, but may be based on a combination of fungal secretions and contributions from other glands of the farming ants.

### The evolution of proteinase activity in the fungal symbionts

Fungi normally produce a wide range of proteolytic enzymes to degrade substrate proteins. The fungal symbionts of lower attines that we investigated (four species from three different genera) had almost exclusively metalloproteinase activity, and virtually no serine proteinase activity. The known phylogenies of attine symbionts [4,33,34] (see also Figure 2) indicate that the lower attine ants rear a paraphyletic group of symbionts that also includes closely related free-living fungi. This implies that we expect these symbionts to have similar enzyme profiles as free-living fungi, which was recently confirmed over a wide range of garden symbionts by De Fine Licht et al. [25]. Our observations thus indicate that the production of metalloproteinases may be an ancestral trait among the attine ant symbionts and suggest that metalloproteinase activity has been evolutionarily conserved while the pH optimum has shifted (or in some cases expanded) from values of ca. 6.0 for the lower attine ant symbionts to values of ca. 5.2 in the higher attine ant and leaf-cutting ant symbionts, which coincide with the acid pH that these ants maintain in their gardens [9,10]. The most parsimonious explanation for these findings is that the free-living relatives of the fungal symbionts would also have proteinases with pH optima of ca. 6, as there seems to be no reason to assume that initial fungus domestication events happened in very acid forest soils. If anything, the average free-living Lepiotaceous fungi prefer mull soils with pH values of at least 6.0 [6]. However, the symbionts of higher attine and leaf-cutting-ants, which have a long evolutionary history as domesticated symbionts, the symbionts of lower attine ants are repeatedly acquired from free-living populations and would thus have had much less time to evolve proteinases with adjusted activity profiles at lower pH.

While metalloproteinase activity appears to be conserved throughout, it appears not to have been upregulated in garden symbionts of basal higher attine ants. The monophyletic group of fungal symbionts reared by *S*. *amabilis*, *T*. cf. *zeteki *and *T*. *sp3*, had reduced metalloproteinase activity and significantly enhanced serine proteinase activity (Figure 2). It has previously been shown that the enzymatic profiles of attine ant symbionts may have a certain amount of plasticity in response to the plant substrate that they grow on [35]. However, differences in the properties of proteinases found in fungal gardens were unlikely to be caused by variations in food substrate composition, as all lab colonies used in the present study were provided with the same leaf material. It seems likely therefore, that the proteinase activity profiles that we obtained have a significant genetic component. Phylogenies of attine ants show that *S*. *amabilis *is more closely related to *T*. cf. *zeteki *than to *T. cornetzi *(T Schultz, pers. comm.), which together with our present results suggests that these ants may have co-evolved with the proteinase activity profiles of their cultivars.

Our quantitative analyses showed that the respective activities of metallo- and serine proteinases in gardens of higher attine ants (including the leaf-cutting ants) tend to be negatively correlated (Figure 1). In most symbionts, the split is very pronounced, with almost complete specialization on one of the two classes of proteinases, although the symbiont of *T. cornetzi *colony 17 is an exception showing almost equal, intermediate activities of the two proteinase classes. This suggests that there may be a trade-off in the expression of proteinases and that there may be adaptive reasons of substrate processing that make the production of either serine- or metalloproteinases most appropriate. Both serine proteinases and metalloproteinases are very widespread in nature and are involved in a wide variety of biological processes. Enzymes belonging to these classes vary significantly in substrate specificity which may correspond to the requirements of fungal ecological niches [36]. One explanation for the shift towards almost exclusive serine proteinase activity might therefore be that the ants that rear these symbionts forage for leaf and flower material that can be more effectively degraded by serine proteinases [37].

Recent studies by Mikheyev et al. [38,34] have shown that North American *Trachymyrmex *rear at least four different species of fungal symbiont, whereas virtually all leaf-cutting ants throughout Latin America appear to rear a single species (*Leucocoprinus gongylophorus *(Möller) (http://www.indexfungorum.org), which has likely been derived secondarily no longer than 2-3 million years ago and swept horizontally through most if not all species of *Acromyrmex *and *Atta *leaf-cutting ants, who themselves had their last common ancestor 8-12 million years ago [39]. Whether this selective sweep had any connection with the symbiont being a strain with upregulated activity of metalloproteinases is presently unknown, but it would be of interest if rare leaf-cutting ants could be found that rear gardens that are more closely related to the serine protease producing *Trachymyrmex *and *Sericomyrmex *symbionts.

We have so far assumed that the measured proteinase activities originate from enzymes produced by the fungal symbiont of the ants. They could also possibly originate from the additional microorganisms found in the fungus gardens of attine ants [40-42]. However, as earlier mentioned [25], the fungal symbiont comprises by far the largest microbial biomass fraction of gardens, so that contributions from other microorganisms should be quantitatively negligible unless they would be specialized symbionts selected for specific enzyme production (for which there is no indication so far). Furthermore, we find consistent patterns of proteinase activity among fungus gardens of the same strain, but differences between strains, which would imply that fungal strains should be obligatorily associated with specific microbial floras if these were to be the predictable sources of the proteinase activities that we measured. Also this would only make sense if these microbial floras would have coevolved symbiont adaptations with specific functions. We cannot exclude that such additional symbionts might exist, but find it hard to base our discussion on such assumption in the absence of any evidence.

### Do leaf-cutting ants suffer from proteinase inhibitors in the leaves that they cut?

Plants produce substantial amounts of proteinase inhibitors to reduce their nutritional value for herbivores [43], who in turn have evolved various mechanisms to circumvent such proteinase inhibitors. As herbivory in attine ants is indirect, it would seem most likely that the ants have come to rely on their fungal symbiont to evolve compensatory measures against proteinase inhibitors, but this may not have been an easy process as the ancestral leucocoprinous fungi that the ants domesticated are leaf litter saprotrophs [6] rather than plant pathogens, and can thus not be expected to have possessed pre-adaptations that enabled them to easily overcome the defense mechanisms present in live plant material.

Putative symbiont adaptations to tackle proteinase inhibitors are unlikely to have arisen in *Trachymyrmex *or *Sericomyrmex *symbionts as these ants mostly use shed flowers and fragments of fallen leaves that are unlikely to be actively defended [37]. Only the most evolutionary advanced leaf-cutting ants, and in particular the genus *Atta, *cut fresh leaves at a large enough scale of defoliation to encounter significant plant defenses by proteinase inhibitors. It would thus be interesting to measure proteinase inhibition in naturally obtained live plant material that *Trachymyrmex*, *Sericomyrmex*, *Acromyrmex *and *Atta *workers provide to their symbionts, to see whether any of these might be specifically targeted towards either serine- or metalloproteinases.

## Conclusions

We have obtained clear indications that the pH optima of proteinases produced by the fungal symbionts of higher attine ants and leaf-cutting ants have become adapted to the acid pH conditions of fungus gardens relative to the surrounding soil. We have also shown that fungus gardens in general have very high pH buffering capacities, and that the production of serine- and metalloproteinases has a distinct phylogenetic pattern, suggesting at least some form of coevolution with the ant farmers. Our data further suggest that trade-offs may exist with respect to the simultaneous production of serine and metalloproteinases across the different species of fungal symbionts. These results are consistent with the symbiosis being constrained by nitrogen availability, due to the low N/C ratio of the plant substrates of fungus gardens [44].

## Methods

There are four main catalytic classes of proteolytic enzymes: aspartic-, cysteine- (thiol-), serine-, and metalloproteinases [45]. It has been inferred that aspartic proteinases are mostly active under acidic pH conditions, that metallo- and serine proteinases usually work optimally under alkaline conditions, and that cysteine proteinase activity is high over a broader range (pH 4 - 7) [46]. We compared both the total and the class-specific proteolytic activity of attine ant symbionts and their free-living relatives across a gradient of different pH conditions.

### Sample material, fungal tissue extract preparation and buffering

Colonies of fungus-growing ants *Apterostigma collare *(nest number Apcol1)*, Myrmicocrypta ednaella *(Myred1, Myred2)*, Mycocepurus smithii *(Mycsmi9, Mycsmi15, Mycsmi32)*, Cyphomyrmex costatus *(Cycos6, Cycos9, Cycos16)*, Cyphomyrmex longiscapus *(Cylon5, Cylon12, Cylon24), *Sericomyrmex amabilis *(Serama7, Serama8, Serama12)*, Trachymyrmex cornetzi *(Trcor1, Trcor3, Trcor4, Trcor10)*, Trachymyrmex sp. 3 *(Trsp3-3, Trsp3-6)*, Trachymyrmex *cf. *zeteki *(Trzet2, Trzet3, Trzet6)*, Acromyrmex echinator *(Acech322)*, Acromyrmex octospinosus *(Acoct367)*, Atta colombica *(Atcol27), *Atta sexdens *(Atsex1), and *Atta cephalotes *(Atcep16) were collected in Gamboa, Panama and maintained under standard laboratory conditions at ca. 25°C and 60 - 70% RH. The ants were supplied with oatmeal (*Apterostigma, Mycocepurus *and *Cyphomyrmex*), oatmeal and fragmented bramble leaves (*Myrmicocrypta, Sericomyrmex *and *Trachymyrmex*) or entire bramble leaves, dry rice and pieces of apple (*Atta and Acromyrmex*).

Strains of non-symbiotic fungi *Agaricus bisporus*, *Pleurotus ostreatus*, *P*. *pulmonarous *and *Lentinula edodes*, which belong to the same fungal order as the leaf-cutting ant symbiont (Agaricales), were obtained from the Department of Mycology and Algology, Moscow State University, Russia. Pure cultures of *Leucocoprinus gongylophorus *were obtained by inoculating mycelium collected from fungus gardens on potato dextrose agar plates and subsequent incubation at 25°C. Fungal cultures were maintained on wort-agar medium and Czapek medium enriched by tryptone (10 g/L) and peptone (10g/L).

Fungi are known to modify environmental pH by producing pH regulating compounds. To detect whether the acidity of fungus garden extracts was due to instantaneous acid production or active buffering, we examined the buffering properties of the extracts. First buffering abilities of the fungal extracts were determined by mixing one μl of fungus garden water extract (1 g in 1 ml) with an equal volume of 0.04 M acid solution (containing phosphoric, boric and acetic acids) or an alkaline solution (0.02 M NaOH), and the resulting pH levels were measured as color changes on pH test paper. The resulting pH change was compared to the pH change obtained using a control acid solution diluted with an equal volume of distilled water, or an alkaline solution two times diluted with distilled water. Next we determined the buffering capacity of the extracts, and compared it to the buffering capacity of extracts made from related non-symbiotic basidiomycete fungi. To measure the approximate buffering capacity of the different fungal species, one gram of fungal biomass or one gram of fungus garden material was homogenized with a pestle in 1 ml of distilled water. Samples were centrifuged (5 min, 5200g) and the supernatant was used for buffer capacity measurements, i.e. the quantity of 1M NaOH that needed to be added to 1 ml the fungus extract in order to change the pH of the suspension by one unit.

### Proteolytic activity assays

Proteolytic activity was measured spectrophotometrically using azocasein (Sigma-Aldrich Co) and the chromogenic p-nitroanilide substrates: Glp-Ala-Ala-Leu-pNa, N-benzoyl-Arg-pNa, and Suc-Ala-Ala-Pro-Phe-pNa (prepared by The State Research Institute of Genetics and Selection of Industrial Microorganisms, Russia). Total and class-specific proteinase activity towards azocasein was tested by determining the rate of hydrolysis after homogenizing pieces of fungus garden material with a pestle in an Eppendorf tube using 2.5 volumes (w/v) of distilled water (in order to keep the natural pH of the sample). Samples were centrifuged at 8000g for 15 minutes and the supernatant transferred to a clean tube. Ten μl of extract was mixed with 15 μl of 2% (w/v) azocasein solution and incubated for 1 hour at 26°C. The reaction was terminated with the addition of 120 μl of 10% TCA after which the suspension was centrifuged for 5 minutes at 14000g and 140 μl of supernatant was added to an equal volume of freshly prepared NaOH (1M). Absorbance was measured at 440 nm using a VERSA_max _microplate reader. Reactions in control samples were terminated immediately after adding azocasein. The difference between treatment and control absorbance (A_440_, at t°C 26°C, 1 hour) was used as a relative measure of enzyme activity. All measurements were performed four times producing means that are presented ± SE.

In order to measure class-specific proteinase activity, the assays were performed in the presence of a protease inhibitor that specifically targets proteases of a certain class. The decrease in activity caused by the inhibitor was used as the class-specific activity value. The inhibition assays were performed using azocasein as described above. 10 μl of sample was preincubated for 3 hours at room temperature with 1 μl of inhibitor resulting in the following final concentrations of the inhibitors (all purchased from Sigma Chemicals Co): For serine proteinase inhibition we used phenylmethane-sulphonul-fluoride (PMSF, 0.57 mM), tosyl lysil chlormethyl ketone (TLCK, 10 μM) and tosyl phenilalanine chlormethyl ketone (TPCK, 10 μM). For cysteine proteinase inhibition we used L-trans-epoxysuccinyl-leucyl-amide-4-guanidino-butane (E64, 5 μM). Activity was also measured after the addition of thyol protecting agent DTT (10mM), which may increase the activity of cysteine proteinases. For metalloproteinase inhibition we used ethylendiaminetetraacetic acid (EDTA, 8 mM) and for aspartyl proteinase inhibition we used pepstatin (2 μM).

Serine proteinases can be further divided into sub-classes based on which substrates they are able to degrade. To determine which sub-classes of serine proteinases were active in fungal gardens, we measured activity towards p-nitroanilides after mixing 5 μl of fungal garden extract, 5 μl of substrate (10mg/ml) and 200 μl of potassium phosphate buffer (0.1M) of pH 5.0 or 7.0 and incubating the reaction mixture at 26°C. The change in absorbance was analyzed using a VERSA_max _microplate reader spectrophotometer at 410 nm. The linear part of the obtained kinetic curve (the dependence of absorbance on time) was used to calculate the enzyme activity.

The effect of pH on total and class-specific proteolytic enzyme activity was measured across a pH range of 3 to 8 (actual measurements at 3.0, 4.0, 5.0, 5.2, 6.0, 7.0, 7.5, 8.0) using 0.2 M Britton - Robinson buffers (A mixture of 0.4 M phosphoric-, 0.4 M acetic-, and 0.2 M boric acid was mixed with different quantities of 0.2 M NaOH to give buffer solutions with the required pH values). The relatively high molarity of the buffers was used to make the natural buffering capacity of the extracts negligible compared to the experimentally induced ones.

To measure the pH dependent proteolytic activity of non-symbiotic fungi, culture fluid of *A*. *bisporus *was used. Modified Czapek medium (0.7 g KH_2_PO_4_, 0.3 g K_2_HPO_4_·3H_2_O, 0.5 g MgSO_4_·7H_2_O, 0.01 g FeSO_4_·7H_2_O, 23.3 g casein in 1 L H_2_O) was inoculated with mycelium from seven days old plated fungus culture and incubated for six days on a rotary shaker (130 rpm, 24°C). Culture liquid was centrifuged (14000g, 20 min) and filtered through filter paper. After adding sodium azide (8% water solution, 2.5 μl to 1 ml of culture liquid) to prevent contamination, fifty μl of culture liquid was mixed with 100 μl of Britton - Robinson buffer (0.1M, pH range from 3 to 8; actual measurements at 3, 4, 5, 5.2, 6, 7, 7.5, 8) and 150 μl of 0.5% water azocasein solution. Reactions were kept overnight (37°C) because of relatively low enzyme activity and then terminated by adding 300 μl of 10% TCA. The reactions were placed at 4°C for 30 min and then centrifuged for 20 min (5200g). 400 μl of suspension was mixed with an equal volume of freshly prepared NaOH (0.5 M) and absorbance at 440 nm was measured using a spectrophotometer (Genesys 10 - UV). The reactions of the control samples were terminated with TCA immediately after adding azocasein. The difference between the absorbance of the treatment and control samples was used as a relative measure of enzyme activity. All measurements were performed three times and presented as means ± SE. Class-specific proteinase activity pH optima were measured in the presence of a protease inhibitors PMSF and EDTA as described above.

Proteolytic activities were finally compared across the different stages of advancement of the symbiosis (lower attine ants, higher attine ants, leaf-cutting ants).

### Phylogenetic analysis

Sequencing of the LSU rRNA gene and the Elongation Factor 1-alpha gene of the fungal symbionts was done by extracting DNA by the Chelex method [47]. Small amounts of fungal tissue were ground in 200 μl of 10% Chelex-100 and heated for 15 min at 95°C. The samples were centrifuged for 3 min at 10,000g after which 1 μl of supernatant was used for PCR. The primer pair LR0R 5'-ACC CGC TGA ACT TAA GC-3' and LR5 5'-TCC TGA GGG AAA CTT CG-3' was used to amplify a fragment of the LSU rRNA gene of about 920 bps, using the following PCR scheme: one cycle of 95°C for 5 min, then 35 cycles of 95°C for 20 sec, 56°C for 30 sec, and 72°C for 1.5 min, ending with one cycle of 72°C for 7 min. The primer pair EF1a-F 5'-GTT GCT GTC AAC AAG ATG GAC ACT AC-3'. [48] and EF1a-R5 5'-CAG GCA ATG TGG GCT GTG TGA CAA TC-3' was used to amplify a fragment of the Elongation factor 1-alpha gene of about 820 bps, using a PCR scheme similar to the one above, although for some of the samples the annealing temperature had to be decreased to 50°C in order to obtain a PCR product. PCR products were sequenced by Eurofins MWG Operon. Nucleotide sequence data are deposited in GenBank with Accession Numbers HQ191224-HQ191277.

The gene sequences were aligned with Clustal W [49], and after deletion of regions that could not be unambiguously aligned, a phylogeny was constructed by maximum-likelihood PhyML-aLRT [50]. The nucleotide substitution model was GTR [51] and the transition/transversion ratios, the proportion of invariable sites and the Gamma distribution parameter were estimated by maximizing the likelihood of the phylogeny. The substitution rate category was set to four, and the input tree to be refined by the maximum-likelihood algorithm was set to BIONJ. The aLRT statistics were performed using the non-parametric Shimodaira-Hasegawa-like procedure. Two of the fungal colonies (Trsp3-6 Trzet6) died during the experiment, so that only the LSU gene could be used for these two samples when constructing the phylogenetic tree.

## Authors' contributions

TAS, JJB, DPH and MS conceived of the study. TAS carried out the protease activity and buffering capacity assays. TAS and MS made the phylogeny. TAS, JJB and MS wrote the manuscript with input from DPH. All authors read and approved the final manuscript.
